# Asian American Female Residents’ Perceptions of Facilitators and Barriers to Leadership in Medicine

**DOI:** 10.1001/jamanetworkopen.2025.12271

**Published:** 2025-05-27

**Authors:** Sarah J. Ho, Jasmine W. Jiang, Catherine T. Yu, Emily Xu, Eunice Y. Yuen

**Affiliations:** 1 Yale School of Medicine, Yale University, New Haven, Connecticut; 2 Department of Psychiatry, Yale School of Medicine, Yale University, New Haven, Connecticut; 3 Yale Child Study Center, Yale School of Medicine, Yale University, New Haven, Connecticut

## Abstract

**Importance:**

When their proportion of faculty positions are accounted for, Asian American women are the most underrepresented group among academic medicine leadership. Despite the importance of diversity and representation in academic medicine leadership, no study to date has explored the specific factors that hinder or support Asian American women’s advancement to leadership roles in academic medicine.

**Objective:**

To characterize perceptions of facilitators and barriers to academic medicine leadership for Asian American women.

**Design, Setting, and Participants:**

In this qualitative study, Asian American female residents were interviewed between December 1, 2023, and April 30, 2024, using an online video platform. Data were analyzed using thematic content analysis. Eligible participants were recruited through word of mouth, social media groups, cold emailing, and snowball sampling. A purposive sampling strategy ensured diversity in ethnicity, geographic locations of past and current institutions, postgraduate years, and specialties.

**Main Outcomes and Measures:**

Key themes related to the perceptions of Asian American female residents on facilitators and barriers to leadership in academic medicine.

**Results:**

Fifteen participants (age range, 25-32 years) who self-identified as female and Asian American (3 [20%] Asian Indian, 6 [40%] Chinese, 2 [13%] Korean, 1 [7%] Punjabi, 1 [7%] Taiwanese, and 2 [13%] Vietnamese) were interviewed. Participants were enrolled in residency programs geographically distributed across the US. Training levels ranged from postgraduate year 1 to postgraduate year 6, spanning 9 residency program specialties. This analysis revealed 4 key themes: (1) role models of leadership, especially the representation of Asian American women in leadership positions; (2) multifactorial development of professional identity, specifically through peers, mentors, and formalized institutional programming; (3) the othering nature of workplaces and institutional cultures, including the exclusionary “boys’ club,” discrimination from patients and colleagues, and burden of familial responsibilities imposed on women; and (4) leadership discordance, including differing ideas of leadership and sociocultural perceptions of Asian American women influencing perceptions of leadership potential.

**Conclusions and Relevance:**

This qualitative study of Asian American female resident physicians’ perceptions of facilitators and barriers to academic medicine leadership found 4 key themes and identified opportunities for intervention, paving the way for enhanced representation of Asian American women in academic medicine leadership.

## Key Points

**Question:**

What are the perceived facilitators and barriers to academic medicine leadership for Asian American female resident physicians?

**Findings:**

In this qualitative study, 15 Asian American female resident physicians were interviewed. Thematic content analysis revealed 4 themes: role models of leadership, multifactorial development of professional identity, the othering nature of workplaces and institutional cultures, and leadership discordance.

**Meaning:**

These results suggest that to address the underrepresentation of Asian American women in academic medicine leadership, interventions need to be implemented on interpersonal, institutional, and societal levels.

## Teaser

This qualitative study assesses perceptions of facilitators and barriers to academic medicine leadership for female Asian American resident physicians.

## Introduction

In 2023, women represented 51.9% of US medical school graduates and 59% of faculty instructors.^1^ However, female representation decreases with each rung in the academic medicine promotion ladder, with women comprising 49% of assistant professors, 43% of associate professors, 29% of full professors, and 25% of department chairs.^1^ Female physicians in academic medicine are consistently underpromoted when compared with their male counterparts,^2^ and men are more likely than women to hold leadership roles within academic medicine even when controlling for differences in academic productivity.^3^ Previous research^3,4^ on the underrepresentation of female physicians in academic medicine leadership implicates multiple factors, including higher rates of attrition for women in academic medicine, family responsibilities, decreased perceptions of self-efficacy and inclusion, and feelings of misalignment between one’s personal values and an institution’s perceived values.

Similarly, although Asian American individuals are well represented among physicians,^5^ Asian American physicians are proportionately the most underrepresented racial group among department chairs relative to their representation among medical school faculty.^6^ Although Asian American faculty represent 20.8% of medical school faculty, they only account for 8.3% of department chairs. In comparison, White faculty represent 71.5% of medical school faculty and 84% of department chairs.^6^ Although Asian American individuals and other groups face some common barriers,^7^ barriers specific to Asian American individuals include racial stereotypes and discrimination regarding social skills and personal characteristics, the model minority myth, and different cultural conceptions of leadership specific to Asian cultures.^8,9,10^ Factors that contribute to the leadership gap for Asian American physicians in academic medicine specifically, however, have not been well studied. Given that Asian American individuals are the country’s fastest-growing racial or ethnic group,^11^ ensuring diversity and representation within academic medicine leadership is crucial because physician leaders drive efforts to improve medicine, health care, and equity at the systems level.

Considering race and gender together, Asian American female physicians are the least represented group among department chair leadership compared with their proportion of overall medical school faculty.^6^ It is important to consider how race and gender intersect^12^ to amplify experiences of inequity and discrimination for Asian American women. Indeed, compared with their Asian American male counterparts and White female counterparts, Asian American female physicians are at highest risk of racial harassment from patients and coworkers,^13^ and Asian American female medical students report a higher rate of microaggressions compared with their Asian American male counterparts.^14^

To our knowledge, there is no existing research about the causes of the disproportionately low numbers of female Asian American physicians in academic medicine leadership positions. In this study, we sought to understand reasons for this discrepancy by interviewing resident physicians because they have been exposed to academic medicine, have begun to actively think about their career plans and aspirations, and represent a target group for early intervention.

Although all participants self-identified as Asian American, we use the term *Asian American* as a broad racial category comprising persons with origins in East Asia, Southeast Asia, or the Indian subcontinent, as defined by the US Census.^15^ This investigation uses semistructured qualitative interviews with female Asian American resident physicians who are considering a career in academic medicine to understand their perceptions of facilitators and barriers to academic medicine leadership.

## Methods

### Study Design

This qualitative study explores the facilitators and barriers to academic leadership through semistructured interviews of Asian American female medical residents. The study was considered exempt from review by the Yale University institutional review board because of the use of deidentified data. Reporting of this study is in accordance with the Consolidated Criteria for Reporting Qualitative Research (COREQ) checklist.^16^ Members of the research team are described in eTable 1 in Supplement 1.

Each participant completed a preinterview survey collecting their demographic information. This survey also included a question asking each participant to specify racial and ethnic categories as represented by the US Census.^15^ Consent to participate was obtained twice for each participant, once electronically at the start of the preinterview survey and once verbally at the start of the interview.

Four authors (S.J.H., J.W.J., C.T.Y, and E.X.) developed an interview guide (eTable 2 in Supplement 1) that consisted of open-ended questions that probed for perceptions of possible facilitators and barriers to leadership in the following domains: individual, interpersonal, communal, institutional, and societal. This structure adapted the socioecological model associated with Bronfenbrenner’s Ecological Systems Theory,^17^ a framework used to understand how different environmental systems come together to impact an individual’s behaviors and development.

Interview questions were reviewed among all authors before the first interview. An outline with example questions was provided to interviewees ahead of time. Interviews were conducted with 1 member of the team (S.J.H., J.W.J., C.T.Y., or E.X.) over video calls on Zoom, version 6.1.11 (Zoom Communications Inc) and were recorded and transcribed. During or after interviews, a noninterviewing team member documented field notes. Interviews took place between December 1, 2023, and April 30, 2024, with interview length ranging from 39 to 88 minutes (median length, 62 minutes). There were no nonparticipants present during interviews. No repeat interviews were required. Transcripts were not returned to participants for checking.

To assess for thematic saturation, a data saturation model approach was used.^18^ Four authors (S.J.H., J.W.J., C.T.Y., and E.X.) met to review and discuss completed interview transcripts and determined data saturation was achieved when the authors identified that no new themes emerged in later interviews.^18^ Achievement of data saturation determined study sample size.

### Participants

Participants were recruited through word of mouth, social media groups, snowball sampling,^19^ and cold emailing. A purposive sampling strategy was used to recruit participants, with special attention to recruiting residents across all postgraduate years (PGYs) and from each US Census region.^20^ To increase the diversity of participants and elicited perspectives, we recruited no more than 2 participants from the same institution and no more than 3 participants within the same specialty. Criteria for inclusion were (1) self-identifying as female and Asian American, (2) being a resident physician at a US academic institution, and (3) having an interest in entering academic medicine. Participants confirmed they met inclusion criteria in the preinterview survey. Interviews were not successfully scheduled for 4 residents who completed a preinterview survey. Participation was voluntary, and no compensation was provided.

### Data Analysis

Two authors (C.T.Y. and E.X.) independently inductively identified codes for each transcript using a grounded theory framework^21^ to describe Asian American female residents’ perceptions of facilitators and barriers to leadership in medicine. Four authors (S.J.H., J.W.J., C.T.Y., and E.X.) discussed, consolidated, and refined the preliminary codes to develop a finalized, combined codebook that was organized hierarchically with child codes cataloged under parent domains: individual, interpersonal, communal, institutional, cultural, and societal.

All transcripts were independently recoded by 2 members of the team (C.T.Y. and E.X.). The same 2 members came together to compare every code in each transcript; all instances of discrepancy between assigned codes were discussed until consensus was reached between the 2 coders. Coding was completed using NVivo, version 14.23.2 41 (International QSR). Based on the combined codebook, study authors (S.J.H., J.W.J., C.T.Y., and E.X.) extracted overarching themes through consensus-based discussion, without participant checking.

## Results

### Demographics

Fifteen female participants (age range, 25-32 years) completed the study. Six ethnic identities were reported: Asian Indian (3 [20%]), Chinese (6 [40%]), Korean (2 [13%]), Punjabi (1 [7%]), Taiwanese (1 [7%]), and Vietnamese (2 [13%]). The geographic locations of participants’ residency programs represented all 9 US Census regions.^20^ Resident training levels ranged from PGY-1 to PGY-6. Interviewees represented 9 distinct residency specialties, with 8 interviewees in nonsurgical fields and 7 interviewees in surgical fields (Table).

**Table.** Participant Demographics

Demographic	No. (%) of participants (N = 15)
Age, y	
25	1 (7)
28	4 (27)
29	5 (33)
30	2 (13)
31	2 (13)
32	1 (7)
Ethnicity	
Asian Indian	3 (20)
Chinese	6 (40)
Korean	2 (13)
Punjabi	1 (7)
Taiwanese	1 (7)
Vietnamese	2 (13)
Geographic location of undergraduate institution^a^	
Pacific	2 (13)
Mountain	1 (7)
West North Central	1 (7)
East North Central	3 (20)
West South Central	4 (27)
Middle Atlantic	1 (7)
South Atlantic	1 (7)
New England	1 (7)
Outside the US	1 (7)
Type of undergraduate institution	
Private	7 (47)
Public	8 (53)
Geographic location of medical school^a^	
Mountain	3 (20)
East North Central	4 (27)
West South Central	3 (20)
East South Central	1 (7)
Middle Atlantic	3 (20)
New England	1 (7)
Type of medical school	
Private	4 (27)
Public	11 (73)
Geographic location of residency program^a^	
Pacific	1 (7)
Mountain	2 (13)
West North Central	1 (7)
East North Central	1 (7)
West South Central	3 (20)
East South Central	2 (13)
Middle Atlantic	1 (7)
South Atlantic	2 (13)
New England	2 (13)
Residency year	
PGY-1	3 (20)
PGY-2	6 (40)
PGY-3	3 (20)
PGY-4	1 (7)
PGY-5	1 (7)
PGY-6	1 (7)
Residency specialty^b^	
Nonsurgical	8 (53)
Surgical	7 (47)

Abbreviation: PGY, postgraduate year.

^a^ Pacific includes Alaska, California, Hawaii, Oregon, and Washington. Mountain includes Arizona, Colorado, Idaho, Montana, Nevada, New Mexico, Utah, and Wyoming. West North Central includes Iowa, Kansas, Minnesota, Missouri, Nebraska, North Dakota, and South Dakota. East North Central includes Illinois, Indiana, Michigan, Ohio, and Wisconsin. West South Central includes Arkansas, Louisiana, Oklahoma, and Texas. East South Central includes Alabama, Kentucky, Mississippi, and Tennessee. Middle Atlantic includes New Jersey, New York, and Pennsylvania. South Atlantic includes Delaware, District of Columbia, Florida, Georgia, Maryland, North Carolina, Puerto Rico, South Carolina, Virginia, and West Virginia. New England includes Connecticut, Maine, Massachusetts, New Hampshire, Rhode Island, and Vermont.

^b^ Nine residency specialties represented.

### Thematic Content Analysis

Four themes emerged through participant interviews. These themes included (1) role models of leadership, (2) multifactorial development of professional identity, (3) othering nature of workplaces and institutional culture, and (4) leadership discordance.

#### Theme 1: Role Models of Leadership

Participants spoke about the importance of seeing role models in positions of leadership (Box 1). In particular, participants expressed that seeing their demographic represented was validating, with one participant stating, “It’s been really helpful to see many women in leadership roles, and I think it’s further encouraging me that in the future, hopefully, when I do pursue this academic route, that more women can be in these leadership roles.” Participants also reported role models as sources of inspiration and guidance for their own career and leadership goals, citing examples such as Asian American female physicians who achieved balance between their career goals and personal lives. Conversely, participants reported that not seeing themselves represented within medicine leadership was a deterrent, with one participant saying, “Simply the absence of prior people in higher positions who look like you can still feel exclusionary, even if no one is overtly being exclusionary.” Examples of role models cited included peers, family members, program directors, and physician leaders encountered at conferences or other networking events.

Box 1. **Illustrative Quotations for Role Models of Leadership**

“It’s been really helpful to see many women in leadership roles, and I think it’s further encouraging me that in the future, hopefully, when I do pursue this academic route, that more women can be in these leadership roles.”
“Simply the absence of prior people in higher positions who look like you can still feel exclusionary, even if no one is overtly being exclusionary.”
“I feel like the Women in Surgery group was extremely validating because you’re seeing what you want to be in 20 years….And all these people talk about their personal lives, too. They talk about them getting married and having kids and prioritizing their life outside of surgery. But they’re still awesome surgeons and awesome at what they do....And then the Asian American [affinity] group–I think that the coolest part about that group is when you listen to these attendings talk, you basically are like, ‘Wow, these people have very similar life experiences to me, and they talk about the same things that I’ve experienced, either the pressure that I felt as a kid or the stereotypes that I felt as a kid’....So I think at the end, it just was more inspiring and more validation [that I could] get through medical school.”

#### Theme 2: Multifactorial Development of Professional Identity

When reflecting on the formation of their professional identities, participants referenced multiple levels of influence. Specifically, 3 subthemes were identified within this theme: (1) peer support, (2) importance of mentorship, and (3) the role of formalized institutional programming. Participants referenced the importance of these interpersonal and institution-level influences in helping them develop their professional identity (Box 2). Participants credited peer support, both through organizations such as affinity groups and through more informal social settings, as being crucial to their professional development (Box 2, subtheme 1). One participant stated about Asian affinity organizations, “You realize it’s not necessarily something wrong with you. It’s something wrong with the system. I think that [Asian affinity groups] give you a better sense of yourself, and it empowers you to do more and gives you more confidence.” Peer support was emphasized through different stages of participants’ education and careers: participants commonly cited the Asian Pacific American Medical Student Association as an impactful affinity group that they participated in as medical students.

Box 2. **Illustrative Quotations for Multifactorial Development of Professional Identity**

**Subtheme 1: Peer Support**

“You realize it’s not necessarily something wrong with you. It’s something wrong with the system. I think that [Asian affinity groups] give you a better sense of yourself, and it empowers you to do more and gives you more confidence.”
“Sometimes when I think I’m the only one going through something, other people are like, ‘Well, yeah, we’re struggling academically, too, or having thoughts about, you know, do I belong in medicine?’ You know, those kind of conversations are important to have, and I think to have connections [and] talk about those things candidly is very empowering and very needed in medicine, regardless of whether you’re a minority or not, but especially if you are a minority and a woman.”
“I’m actually really grateful to my experience in APAMSA, because it showed me that no matter what stage of training you’re in, no matter what experiences you have, what city you’re in, what institution you’re at, there are opportunities to continue climbing up and getting leadership experiences....What they taught me was to just take the experiences as they come [and] make sure it’s something that you are passionate about and that’s within your values.”

**Subtheme 2: Importance of Mentorship**

“I’ve been really fortunate having really amazing mentors in my life, and they’ve really encouraged me to pursue leadership positions. They’ve really helped advocate for me, writing letters of recommendations [and] providing opportunities….I think that support has been really beneficial for me, and I think it has not only helped develop my understanding of leadership, but I think it has also helped me feel more confident in really trying to embrace that role as well.”
“I don’t believe I would have been able to make it this far if it weren’t for that mentorship, other women who have faced similar barriers but have now made it to these academic leadership roles, who are advocating for me and rooting for me. I think that’s what has made it a lot easier to be able to join these leadership roles and committees.”

**Subtheme 3: Role of Formalized Institutional Programming**

“Going back to my medical school, I really did appreciate the DEI program, the fact that they connected me with different faculty members who were primarily women of color in leadership roles. They became my mentors. They gave me these opportunities. It gave me networking skills but also that leadership quality as well. And I think it really helped me further develop those skillsets.”
“[As someone who is] first generation in medicine, [having information about] those opportunities that other people come in knowing about would be great…it’s that quote unquote hidden agenda of what you’re supposed to be accomplishing that [should be] more openly available or openly talked about.”
“[Having a mentoring system or an advising system] would really help residents to feel like they are able to pursue leadership, because they [would] know who to talk to, instead of the resident trying to use all their limited amount of free time between sleep [and] just surviving residency.”

Abbreviations: APAMSA, Asian Pacific American Medical Student Association; DEI, diversity, equity, and inclusion.

In addition, participants emphasized the role that mentors had in guidance and active sponsorship (Box 2, subtheme 2). These mentors offered career advice and simultaneously served as sponsors, taking an active role in advocating for their mentees and connecting them with leadership opportunities to further their career. Some participants noted that they sought out mentors who were Asian American and female when possible and found that they more easily related to mentors who had similar identities and experiences to their own. Other participants stated that the identities of their mentors, and sometimes even their mentors’ specialties and positions, were less relevant; more relevant was their accessibility, communication style, and advocacy on behalf of their mentee.

Lastly, many participants expressed the importance of formalized institution-level programming in preparing residents to pursue leadership roles within medicine (Box 2, subtheme 3). Some participants reported that their institution’s existing programs were crucial in their leadership development, whereas others were more equivocal about whether institutional diversity, equity, and inclusion (DEI) programs were effective generally or inclusive of Asian American female residents. Many other participants noted that their institution did not have any formalized programming on leadership development for resident physicians and expressed the desire for more formalized education on areas such as giving and receiving feedback, conflict resolution, and networking.

Participants noted the self-initiative needed to seek out professional connections, given the lack of institutional programming and the many responsibilities and time constraints of resident schedules. One participant stated, “[Having a mentoring system or an advising system] would really help residents to feel like they are able to pursue leadership, because they [would] know who to talk to, instead of the resident trying to use all their limited amount of free time between sleep [and] just surviving residency.” The need for intrinsic motivation was emphasized by participants, especially those who were the first in their family to enter medicine or go to school in the US. These participants endorsed lacking knowledge about how to navigate mentorship, networking, and career planning, especially in comparison to classmates with existing personal connections within medicine.

#### Theme 3: Othering Nature of Workplaces and Institutional Culture

Across interviews, participants described feeling othered in their workplaces and negative institutional culture as deterrents to pursuing academic medicine in general or leadership in academic medicine (Box 3). Many participants discussed microaggressions and macroaggressions that they experienced from other health care practitioners and patients, wherein microaggressions are subtle acts that carry a derogatory or discriminatory meaning,^22,23^ and macroaggressions encompass intentional and unconcealed discriminatory acts.^24^ Participants cited being confused for other female Asian residents by other members of the health care team and being treated differently than male residents by attending physicians: “This attending will not call me a doctor, but he’ll call the male residents ‘physicians’ in front of staff members as well as patients” (Box 3). Many participants noted having to contend with discrimination and harassment from patients: “[Patients] don’t treat you with as much respect. The first question out of everyone’s mouth is, ‘Where are you from? Are you from here? Are you from India?’” (Box 3).

Box 3. **Illustrative Quotations for the Othering Nature of Workplaces and Institutional Culture**

“It’s like the hierarchy. It’s your Caucasian male physicians, and then it’s your female Caucasian physicians, and then us Asian American [female physicians] are kind of lower down….[Patients] don’t treat you with as much respect. The first question out of everyone’s mouth is, ‘Where are you from? Are you from here? Are you from India? When are you going back to India to visit?’”
“I’ve had attendings…[who] won’t use my doctor title….This attending will not call me a doctor, but he’ll call the male residents ‘physicians’ in front of staff members as well as patients…I’ve also had experiences where patients will make very crude sexual comments, and there is some harassment there as well.”
“We have one other Asian female resident in this hospital, and people get us confused all the time…we unfortunately have the same last name, and we get put into each other’s cases. People confuse us in the hallways, people are like, ‘Oh, you were just here.’ No, I wasn’t.”
“Oftentimes they’ll be like, ‘Oh, yeah, we just wrote up the guidelines while sitting in an airport...because we went on a trip together’...and it’s just who happened to be in the room at that time. I always joke about wanting to learn how to play golf….[Men] are always trying to play golf with each other, and I’m like, ‘Well, I want to be in the room [too] to network and stuff.’ But it’s just the thing with male-dominated fields, like it’s hard to break into that.”
“Making leadership and roles more accessible for women…maternity leave, child support, those sorts of things, will relieve the burden of ‘life things’ that tend to happen with us at the same time. Our reproductive years are the years that we’re doing training. Trying to work on resources and ways to support that aspect of things will then free up Asian American women for career things that they are probably pretty passionate about but just don’t have the time and energy to go do.”

Participants spoke about the exclusionary culture of academic medicine, with one participant referring to the leadership at their institution as a “boys’ club.” Participants noted that academic medicine did not seem to be responsive to or inclusive of personal priorities that female physicians often juggle more so than male physicians. For instance, participants spoke of inadequate institutional policies about maternity leave, breastfeeding, and childcare.

#### Theme 4: Leadership Discordance

When asked about their perceptions of leadership, some participants indicated that societal views of the ideal leader were incongruent with their personal ideas of what leadership in academic medicine should be (Box 4, subtheme 1). Participants stated that current conceptions of leadership within academic medicine were focused on research and publication count and less focused on advocacy and community health efforts. Many participants also noted a pattern of women taking on extra “soft skills” work that was usually uncompensated and unrecognized, such as organizing program social events and participating in applicant recruitment efforts. Some participants also stated that their culturally informed ideas of leadership did not align with the way that their institutions perceived leadership, with one participant mentioning that her culture portrays leadership as “acts of kindness that are done in silence” as opposed to being “outspoken” and “having the ability to command.”

Box 4. **Illustrative Quotations for Leadership Discordance**

**Subtheme 1: Different Ideas of Leadership**

“Something that’s kind of missing in academic medicine institutionally is that…community engagement is not necessarily acknowledged as an area of professional endeavor…there are a lot of physicians who are part of it and doing those things as part of their career, but there’s not a structural way to acknowledge that as an achievement.”
“I feel like women are always doing the free work that’s unpaid. You don’t get hours for the number of meetings that you do on a Monday at 7 pm. In academics, the currency is publications and how many grants can you get....I’m often volunteering to lead a lot of these [residency meet and greets]. But they’re tiring...it can be frustrating to see the men not signing up for these things and giving up their time….And it’s just the women who are showing up because we’re trying to make sure to recruit people who are also like us and are going to be good co-workers.”
“I think leadership can be different depending on where you’re from, how you’re raised, how you’re brought up. For example, in Asian culture and in my Vietnamese family, I think leadership is about service, about taking care of other people, and doing what needs to be done. Often, [leadership] can be acts of kindness that are done in silence, and it’s not really about being vocal. I think in academic medicine [or just in American society in general]…leadership is defined [by] being outspoken and having the ability to command.”

**Subtheme 2: External Perceptions of Leadership Capacity**

“There’s this gender and racial discrimination at play…[Asian American women] are stereotypically seen as quiet and submissive. And so there’s this preconceived notion that we can’t be leaders. But then the flip side here is, if we start speaking up and talk about our opinions, we’re constituted as ‘the dragon lady.’ And so we’re seen as bossy and unlikable. It becomes really quite difficult for us to be given and provided these opportunities for leadership because of these stereotypes.”
“[With being an] Asian American female, I feel like there’s this intersectionality in the sense that there are a lot of stereotypes in terms of how you should act….You’re not necessarily supposed to be direct…you’re more gentle and submissive is the expectation. And then when you’re not that, it can be taken the wrong way. Just on the topic of Asian American identity more broadly, I think there’s also this perception that you are hard workers, kind of like a model minority stereotype where you’re able to get the work done, get it done well, get it done efficiently, but no one ever actually thinks of you as necessarily leadership material. You’re more like a worker bee. But you’re never the...queen bee.”
“People hate to see an Asian woman succeed. They can find all sorts of flaws. ‘She’s bitchy, she’s catty, she’s a hard ass, she’s whatever.’ And again, you put all those attributes in a man, and they’re like, ‘Oh, he works so hard, he’s so detail-oriented. He cares so much. He’s passionate.’”

**Subtheme 3: Self-Perception of Leadership Capacity**

“If your patients and people you work with always feel like you’re less than in some ways than a tall White male person, then that definitely doesn’t help your confidence and your ability to take on those additional roles and responsibilities.”
“[There is a] pressure of continuing Asian traditions...and balancing that with, ‘Do I even want to have children? Is it possible to have children in fellowship? Is it possible to pursue my career while trying to navigate whether I want to start a family? When am I going to get married?’”

Participants also expressed that sociocultural perceptions of Asian American women had affected how others had viewed their leadership capacity (Box 4, subtheme 2). The combination of negative gender stereotypes and negative stereotypes of Asian American individuals resulted in the perception of Asian American women being “quiet and submissive” and “docile.” Moreover, participants noted a cost to challenging these biases because they were subsequently perceived as “bossy and unlikable,” “catty,” and “the dragon lady” when advocating for themselves, with “dragon lady” being a name that reflects stereotypes that lie at the intersection of being both a woman and Asian American. Regarding perceptions of Asian American individuals more broadly, one participant reflected that an Asian American individual was more likely to be perceived as “a worker bee” rather than as a leader.

In some cases, societal perceptions of Asian American women affected how participants viewed their own leadership capacity (Box 4, subtheme 3), with one participant saying, “If your patients and people you work with always feel like you’re less than in some ways than a tall White male person, then that definitely doesn’t help your confidence and your ability to take on those additional roles and responsibilities.” In addition, some participants reported that sociocultural pressures in their personal lives affected how they thought of their career goals. Cultural and familial expectations about the role of women in the household and the importance of finding a partner and starting a family were noted as stressors. Participants endorsed uncertainty about what to prioritize when thinking about their future and how they might balance their professional and personal lives.

## Discussion

This study illuminates 4 themes underlying the disproportionately low representation of female Asian American physicians in academic medicine leadership: role models of leadership, multifactorial development of professional identity, the othering nature of workplaces and institutional culture, and leadership discordance. Our findings explore the intersection of race and gender in the context of academic medicine leadership and add to previous literature that has examined the experiences of women^25,26^ and Asian American people^27,28^ in medicine.

Our findings highlight facilitators of Asian American female leadership within academic medicine. Participants underlined the importance of mentorship and role modeling in academic medicine and physician leadership development during the residency training period, aligning with areas of intervention identified in previous literature.^29,30,31,32,33,34^ Participants also found support in affinity groups, informal peer groups, and professional organizations focused on Asian American women. On the other hand, participants identified many barriers to academic medicine leadership. Participants reported feeling othered, experiencing discrimination, and being labeled with stereotypes. They also stated that their work in DEI efforts and community bonding was often undervalued and that their own ideas of leadership were not compatible with current leadership paradigms. Participants also discussed feelings of competing sociocultural and familial expectations when thinking about career planning.

Our findings agree with previous research that highlights the challenges that women and people of color encounter in medicine.^35,36,37,38,39,40^ Prior research has found that women and faculty of color must undertake “invisible” labor in academia,^41,42^ often linked to diversity initiatives, social events, or recruitment efforts; our findings indicate that this phenomenon is present for Asian American female resident physicians and may serve as a deterrent to pursuing leadership. Participants also emphasized the othering nature of male-dominated spaces, aligning with previous work.^43^

Importantly, Asian American female resident physicians encounter challenges that are specific to their intersectional identities, such as stereotypes of docility and passivity.^44,45,46,47,48^ Participants were commonly perceived as “quiet and submissive” and “a worker bee,” negative stereotypes that result from the compounding effects of gender and racial discrimination and in turn undermined external or self-perceptions of leadership potential. Simultaneously, participants noted the double bind they had to contend with, wherein not conforming to these stereotypes led to being perceived instead as “bossy and unlikable,” “catty,” and “the dragon lady,” perceptions that evoke distinct racial and gendered stereotypes of their own. Participants also reported sociocultural and familial expectations of Asian American women as family oriented, similar to what has been previously documented.^44,46^

Broadly speaking, a cultural change needs to occur wherein Asian American women, resident physicians and otherwise, are not judged by whether they fit a stereotype but by their character and competency. Although large-scale change can take substantial time, immediate and tangible actions can be taken. On the basis of the findings of this study, we encourage institutions to be more inclusive of different leadership qualities and to more intentionally consider advocacy, education, and community building as facets of leadership along with other metrics of scholarship and clinical work. Thinking about leadership more expansively also requires that institutions change their policies so that Asian American female physicians face fewer obstacles to leadership, such as improving maternity leave and child support policies and building an institutional culture that does not disadvantage them. Faculty training in diverse leadership styles and recognizing implicit bias^49^ may be beneficial as well. The results of this study also emphasize the importance of effective peer support and active and engaged mentorship for the empowerment of Asian American female physicians. Intentional efforts from individuals, organizations, and institutions to foster peer and professional communities as well as formalized mentorship programs should be undertaken to provide pathways to leadership and professional growth. In addition, institutions and professional organizations supporting Asian American female physicians should develop and implement curricula on career planning and leadership development as a part of residency training. Existing leadership development programs for resident physicians have shown evidence of positive outcomes^50^ and can serve as models for other institutions to develop similar programs.

Our proposed interventions serve as stepping stones toward a greater goal of equity and representation in medicine and hopefully will work to support other marginalized groups within academic medicine. Recent policy changes have pressured institutions to pull back on such initiatives^51^; however, these interventions remain critical and actionable to address gaps in representation within academic medicine leadership. The increased visibility of Asian American women in leadership will hopefully combat existing negative stereotypes and positively influence society’s perceptions of what a leader can look like. As for the individual, we hope that Asian American female physicians can recognize their unique strengths and experiences and potential for effective leadership.

### Limitations

This study has limitations. Although we sought to maximize geographic and ethnic diversity, recruiting participants from all regions defined by the US Census and representing 6 ethnic identities, the perspectives of 15 participants do not encapsulate the diversity of perspectives of all Asian American female resident physicians. Indeed, we acknowledge that our study does not account for the variability and nuances that exist among different ethnic subgroups, generational statuses, and other sociocultural statuses that may impact an individual’s experiences. Additionally, although participants did not need to have a specific interest in leadership within academic medicine, the study’s title may have attracted those with such an interest, potentially skewing the sample toward individuals inclined to pursue leadership roles. Future studies may also consider interviewing early or midcareer faculty who may have more experience in academic medicine and who may be more concretely considering leadership opportunities. Finally, our study does not focus on impacts of other factors, such as training in surgical vs nonsurgical specialties, male-dominated specialties, or locations with predominantly White populations, which can be explored in further studies.

## Conclusions

In this qualitative study of Asian American female resident physicians’ perceptions of facilitators and barriers to academic medicine leadership, we found that exposure to role models, peer support, effective mentorship, and formalized institutional programming are facilitators to leadership. An exclusionary workplace culture, incidences of discrimination and stereotyping, differing ideas of leadership, and sociocultural expectations, influenced by the intersectionality of gender and race, were reported as barriers. These findings identify opportunities for interventions at the interpersonal, organizational, and institutional levels that may enhance the representation of Asian American women in academic medicine leadership.

## Notes

Supplement 1. **eTable 1.** Consolidated Criteria for Reporting Qualitative Research (COREQ) Domain 1: Research Team and Reflexivity **eTable 2.** Interview Guide

Supplement 2.
Data Sharing Statement
