# Supplementary material for: Asian American Female Residents’ Perceptions of Facilitators and Barriers to Leadership in Medicine
Source: JAMA Netw Open. 2025 May 27;8(5):e2512271. doi: 10.1001/jamanetworkopen.2025.12271 (PMC12117468; doi:10.1001/jamanetworkopen.2025.12271)
Supplement: Supplement 1. — eTable 1. Consolidated Criteria for Reporting Qualitative Research (COREQ) Domain 1: Research Team and Reflexivity eTable 2. Interview Guide [file jamanetwopen-e2512271-s001.pdf]

Supplemental Online Content

Ho SJ, Jiang JW, Yu CT, Xu E, Yuen EY. Asian American female residents' perceptions of facilitators and barriers to leadership in medicine. *JAMA Netw Open*. 2025;8(5):e2512271. doi:10.1001/jamanetworkopen.2025.12271

eTable 1. Consolidated Criteria for Reporting Qualitative Research (COREQ) Domain 1: Research Team and Reflexivity

eTable 2. Interview Guide

This supplemental material has been provided by the authors to give readers additional information about their work.

© 2025 Ho SJ et al. *JAMA Network Open*.

eTable 1. Consolidated Criteria for Reporting Qualitative Research (COREQ)
Domain 1: Research Team and Reflexivity

Interviews were conducted by C.T.Y., E.X., J.W.J., and S.J.H., who are all medical students. Author E.Y.Y. is an attending child and adolescent psychiatrist and is an Assistant Professor of Psychiatry. All study authors identify as female. Senior author E.Y.Y., as well as J.W.J. and E.X., had prior experience with qualitative research methods. There were no established relationships between participants and authors prior to study commencement. Participants were informed of interviewing authors' stage of medical training.

© 2025 Ho SJ et al. *JAMA Network Open*.

eTable 2. Interview Guide

Hello. Thank you for joining us today! As a reminder, we are conducting a qualitative study to understand the perspectives of Asian American female residents on leadership in academic medicine. In today's interview, we will spend approximately 1 hour together. We will ask you a series of questions about leadership and ask that you frame your responses to the questions in the context of academic medicine and in reference to yourself and your personal experiences. Your participation in this interview is voluntary, and you do not have to answer any questions you don't want to answer. Importantly, there are no right or wrong answers; we are here to learn from you and your experiences and thoughts.

Introductory

- Can you start by speaking about where you grew up and your upbringing? Can you talk about the diversity of the area you grew up in? (Probe for Asian American representation) Where did you go to college and medical school?
- What are your career goals in academic medicine?
- What does leadership in academic medicine mean to you?
- Do you envision leadership as being part of your career in academic medicine? Why or why not?

Leadership/Intrapersonal

- What qualities and/or skills do you think are necessary to possess a leadership position?
- Where do you see yourself stand in terms of possessing these qualities and/or skills?
- What do you think has contributed to your sense of yourself as a leader?

Interpersonal

- Have you had any personal relationships that have influenced your ideas about leadership, particularly about leadership in academic medicine?
- *If not mentioned:*
    - How has your family influenced your career goals and attitudes toward leadership in academic medicine? Do you have any family in medicine?
    - *If not mentioned*:
        - Have your parents played a role?
        - Have any partners played a role?
    - Have you had any mentors across your undergraduate institution, medical school, or current residency program?
        - *If yes:* How did they impact your career goals, if at all?
        - *If no*: Why not?
    - How have interactions with your peers and/or friends affected your career goals and how you thought of leadership in academic medicine?
- Is there anyone else you can think of who has been important or influential in how you think about leadership and leadership in medicine?

Community

- Have you been part of any organizations supporting Asian Americans and/or women? Any other affinity groups?
    - *If yes*: How have they impacted your sense of self in academic medicine and leadership? How have they impacted your career goals?
    - *If no*: Could you please elaborate on why not?
    - Do you think these kinds of organizations are helpful or useful?

© 2025 Ho SJ et al. *JAMA Network Open*.

- Can you think of any other communities or groups that have impacted how you think about leadership?

Institutional

- What features or programs about your current or past institutions do you perceive as facilitators for academic medicine leadership?
- What features of your current or past institutions do you perceive as barriers to academic medicine leadership?
- How do you think your identity as an Asian American woman has impacted your perception of the facilitators/barriers that you've just mentioned?
- Have you been aware of DEI (diversity, equity, and inclusion) efforts at any of your institutions, either in the past or currently? How have they impacted your professional goals, if at all?
- What institutional features or programs are missing that you think are important for your career and leadership goals?

Societal

- How do you think sociocultural expectations of Asian American women have influenced your training and professional goals?
- Have you ever experienced discrimination in an academic medicine setting?
    - *If yes*: How has it impacted you?

Other general/conclusion questions

- There are disproportionately low numbers of Asian Americans, especially Asian American women, who are represented among academic medicine leadership. Why do you think this is?
- What can Asian American women bring to the table?
- What do you think could be done to increase representation?
- What do you wish to know about leadership at your stage of career?
- Is there anything else you would like to talk about that we have not yet mentioned?

© 2025 Ho SJ et al. *JAMA Network Open*.
