# Supplementary material for: Asian American Female Residents’ Perceptions of Facilitators and Barriers to Leadership in Medicine
Source: JAMA Netw Open. 2025 May 27;8(5):e2512271. doi: 10.1001/jamanetworkopen.2025.12271 (PMC12117468; doi:10.1001/jamanetworkopen.2025.12271)
Supplement: Supplement 2. — Data Sharing Statement [file jamanetwopen-e2512271-s002.pdf]

Data Sharing Statement

Ho. Asian American Female Residents' Perceptions of Facilitators and Barriers to Leadership in Medicine. *JAMA Netw Open*. Published May 27, 2025.
doi:10.1001/jamanetworkopen.2025.12271

Data
Data available: No

Additional Information
Explanation for why data not available: Data will not be made available in order to preserve participant anonymity.
